# Challenges in Forecasting Antimicrobial Resistance

**DOI:** 10.3201/eid2904.221552

**Published:** 2023-04

**Authors:** Sen Pei, Seth Blumberg, Jaime Cascante Vega, Tal Robin, Yue Zhang, Richard J. Medford, Bijaya Adhikari, Jeffrey Shaman, for the CDC MIND-Healthcare Program

**Affiliations:** Columbia University, New York, New York, USA (S. Pei, J. Cascante Vega, T. Robin, J. Shaman);; University of California, San Francisco, California, USA (S. Blumberg);; University of Utah, Salt Lake City, Utah, USA (Y. Zhang);; University of Texas Southwestern Medical Center, Dallas, Texas, USA (R.J. Medford);; University of Iowa, Iowa City, Iowa, USA (B. Adhikari)

**Keywords:** antimicrobial resistance, infectious disease forecasting, healthcare-associated infections, *Suggested citation for this article*: Pei S, Blumberg S, Cascante Vega J, Robin T, Zhang Y, Medford RJ, et al. Challenges in forecasting antimicrobial resistance. Emerg Infect Dis. 2023 Apr [*date cited*]. https://doi.org/10.3201/eid2904.221552

## Abstract

Antimicrobial resistance is a major threat to human health. Since the 2000s, computational tools for predicting infectious diseases have been greatly advanced; however, efforts to develop real-time forecasting models for antimicrobial-resistant organisms (AMROs) have been absent. In this perspective, we discuss the utility of AMRO forecasting at different scales, highlight the challenges in this field, and suggest future research priorities. We also discuss challenges in scientific understanding, access to high-quality data, model calibration, and implementation and evaluation of forecasting models. We further highlight the need to initiate research on AMRO forecasting using currently available data and resources to galvanize the research community and address initial practical questions.

Antimicrobial resistance (AMR) is a leading threat to global health (*1*). An estimated 4.95 million deaths were associated with bacterial AMR in 2019 worldwide (*2*), mostly caused by 6 pathogens: *Escherichia coli*, *Staphylococcus aureus*, *Klebsiella pneumoniae*, *Streptococcus pneumoniae*, *Acinetobacter baumannii*, and *Pseudomonas aeruginosa*. To limit the spread of antimicrobial-resistant organisms (AMROs) and reduce AMR-related disease burden, improved predictive intelligence is required to better estimate the emergence and spread of AMR within populations and healthcare facilities. However, efforts to operationally forecast the burden of AMROs (i.e., for real settings in real time) are not active as of January 2023. 

Real-time infectious disease forecasting aims to generate estimations of future disease incidence at the population or community level, which can be subsequently evaluated using the observed outcomes. During 2010–2020, predictive models for viral and acute infectious diseases such as influenza (*3*), dengue (*4*), and COVID-19 (*5*) have been put in place and tested in the real world. In contrast, no predictions have been generated and validated for AMROs. Here, we discuss the potential for AMRO forecasting at the population and facility scales, highlight challenges for this field, and suggest future research priorities.

## Mathematical Modeling of AMR

Mathematical and statistical models have contributed to the fight against AMR (*6*) and may enable predictions of AMR at different scales. Modeling studies of AMROs have been undertaken to clarify the factors associated with AMR at the population scale. For example, time series analyses have been used to quantify the association between antimicrobial use and prevalence of resistance in populations (*7*,*8*). Such analyses are valuable because they typically result in a set of coefficients representing the effect of antimicrobial use on future AMR outcomes; however, those models do not project AMR prevalence forward and rigorously evaluate future predictive accuracy. In parallel, process-based mathematical models have been developed to study the transmission of AMROs (*9*–*11*), simulate the competition between resistant and sensitive strains (*12*–*16*), and evaluate the effects of various policies (*17*–*22*). More recently, detailed individual-level models informed by historical patient movement or contact with healthcare workers have been used to represent transmission networks and heterogeneity in healthcare facilities (*23*–*26*). Those modeling studies have enriched understanding of the evolutionary dynamics of resistance and AMRO transmission dynamics but have not been used to produce operational AMR predictions.

## Forecasting AMROs at Different Scales

Operational forecasting of AMR could have implications for public health and patient care. Depending on the intended use, AMRO forecasting can be done at population-level and facility-level scales. Forecasting at the population level aims to predict the trend of infection or carriage prevalence in the general population for relatively long periods of months to years. For AMR pathogens, the forecast target might be the number of AMR infections or the proportion of isolates exhibiting resistance. Those predictions would estimate future AMR burden (e.g., deaths, hospitalization, days of work lost, or direct and indirect economic costs) and the evolution of resistance. If used in real time, those predictions would support situational awareness and inform public health policies such as antimicrobial drug stewardship and more targeted antimicrobial prescription guidelines to slow down AMR spread.

At the facility level, the forecast target of interest might be the number of AMR infections with clinical symptoms within a hospital or hospital system. Such predictions would support control of nosocomial AMRO transmission and resource planning for equipment, medications, staffing, and space in response to potential patient surges. Depending on the clinical relevance, the forecast horizon might be days or months. Of note, predictive models connecting multiple healthcare facilities in a region could elucidate the risk for AMR introduction through interhospital patient transfer and support decision making for preemptive measures in facilities without ongoing transmission.

## Challenges in AMRO Forecasting

Although models and data differ considerably for forecasts at various scales, some common challenges impede the development and operational use of predictive models for AMR. Here we summarize these issues and highlight several research priorities to address these challenges in future studies.

### Scientific Understanding

For forecasting using mathematical and statistical models, it is critical to understand the key processes affecting AMR spread. Those processes are often represented as nonlinear effects in forecasting models and, if not properly specified, will produce forecasts that quickly diverge from the truth. As of 2023, many questions on AMR remain open (*27*). For instance, the role of antibiotic use in driving AMR is not fully understood, particularly the effects of co-selection (i.e., selection of resistance that is broader than the specific target of an antimicrobial prescription) (*28*) and the relationship between outpatient use of antimicrobial drugs and resistant infections of hospitalized patients. More generally, it is not yet known which type of antimicrobial drug use (e.g., community use, hospital use, or veterinary use) has the greatest effect on AMR emergence (*29*). After the emergence of AMROs, it is unclear how competition with susceptible strains affects the incidence of resistant strains and how to explain their coexistence over long time periods (*30*). Likewise, the issue of spillover (i.e., transmission of AMR across locations) is arguably a substantial challenge for forecasting that has not been addressed (*31*).

In healthcare facilities, it is unknown how contact networks and heterogeneity of exposure to antibiotics shape the spread of AMR; it is hard to disentangle the roles of community importation and nosocomial transmission; and it is difficult to quantify the relative transmissibility among classes of persons (patients, healthcare workers) and the environment. In addition, individual-level causal relationships between the type and duration of therapy and resistance emergence remain unknown in most instances. The human microbiome serves as a reservoir of antimicrobial resistance (*32*–*34*); however, many outstanding scientific questions on microbiome effects are still under active research as of January 2023. Further studies are needed to examine the role of bystander selection (i.e., selection of resistance on microbes that are not the target pathogen) in AMR emergence (*35*,*36*), the reason treatment with cephalosporins is a risk factor for vancomycin-resistant *Enterococcus* colonization (*37*), and the difference between detectable colonization and high-level colonization.

To date, infectious disease forecasting has primarily focused on acute viral infections for which the pathogen and its disease or clinical outcome can be directly linked. For instance, viral load is generally correlated with infectivity and disease phenotype (mild to severe) and, therefore, with illness and death rates. Those correlations make definition of the forecasting target (e.g., incident rates of cases, hospital admissions, or deaths) relatively straightforward. However, for bacterial or fungal species, relationships between pathogen load and clinical outcomes are unclear. Because many bacterial species are commensal with their human host and have varying probabilities of presence across body sites, it is challenging to definitively determine whether a person is colonized. Without accurate observation of colonization, AMR burden is not well resolved and, consequentially, is more difficult to forecast.

### Accessing High-Quality Data

Forecasting is fundamentally a data-driven task. Without sufficient data, predictive models cannot be properly trained and evaluated. As of 2023, data that can inform operationally useful forecasts of AMR remain scarce. At the population level, several surveillance systems do exist. For instance, the US National Antimicrobial Resistance Monitoring System for Enteric Bacteria tracks changes in antimicrobial susceptibility for certain enteric bacteria in ill persons, retail meats, and food animals (*38*). However, consistent long-term records of AMR pathogen profiles are lacking in most countries, particularly in low- and middle-income countries and for emerging AMROs with limited cases (*39*). In addition, several major pathogens responsible for healthcare-associated infections have not been included in surveillance.

At the facility level, AMR data from EHR have become increasingly available to researchers in recent years. In healthcare settings, more attention has been given to infected patients with clinical manifestations. Surveillance for asymptomatic AMRO carriage is not prioritized because it is not of immediate clinical interest, although such carriers play an important role in onward transmission (*20*). Such incomplete observation hinders estimation of overall AMRO prevalence and may lead to biased prediction targets. In addition, data on nonbiologic processes driving AMR pathogen transmission, such as patient behavior and interactions with healthcare workers, are difficult to collect. In cases for which relevant data are available, data quality may be poor because records can include errors and misclassification. Even for structured EHR data, both predictive variables and outcomes (e.g., colonization) can suffer from missing data.

### Model Calibration

Model calibration is the process by which a mathematical model is tuned to reproduce empirical observations. Although this process does not guarantee accurate prediction, model calibration provides an initial check that the model can closely replicate historical data. Studies that calibrate AMR models to empirical data have been published (*23*,*24*,*40*–*42*). However, as the structure of AMR models becomes increasingly complex, computational difficulties arise in fitting these models to observations of different types and at various scales. For instance, population-level prevalence, individual-level test results, and genomic sequences of pathogens convey different pieces of information on AMRO transmission, and calibrating AMR models to these observations simultaneously is a challenge. AMRO transmission is intrinsically stochastic with large uncertainty. Quantifying the uncertainty of predictions generated by complex AMR models is difficult, especially for models that track individual persons and their contacts. Calibration approaches, and their success, usually depend on the specific model construct and the form of observations.

### Implementation and Evaluation

One prominent challenge for AMRO forecasting is the operational implementation and prospective evaluation of predictive models (i.e., generating forecasts in real time and evaluating those forecasts once prediction targets are observed). There are no guidelines on such implementation for AMRO forecasting, such as appropriate data collection and forecast targets. Questions remain open on the proper time scale of forecast horizon, the frequency at which models need to be updated, and the fundamental limit of predictability of models. For long-lead forecasting, evaluation requires data collection in a consistent manner over a long time period. In healthcare facilities, the practice of testing and reporting AMR infections may change over time, which further complicates using such data records and forecast evaluation. A collaborative effort that standardizes training datasets, forecast targets, forecast horizons, and proper scoring rules for evaluating forecast performance (e.g., the FluSight influenza forecasting challenge [*43*–*45*], the dengue forecasting challenge [*4*], and the RAPIDD Ebola forecasting challenge [*46*]) can potentially stimulate advances in operational AMR forecasting.

A particular challenge for implementing AMRO forecasting is to handle uncertainty in predictions; uncertainty exists because of imperfect data and a notable degree of variability in many AMR-related processes. Quantifying such uncertainty is critical in other predictive fields, such as numerical weather prediction. For AMROs, whether at the facility level (e.g., determining which patients need to be on contact precautions) or the community level (e.g., public health officials making recommendations for prescribing guidelines because of AMR), decision makers must make decisions that leverage uncertain information. This truism holds for observations as well as forecasts. Designing optimal decision frameworks and architectures that best use forecasts, given their uncertainty, is a needed long-term goal.

Effective communications between modelers and stakeholders such as public health officials, healthcare institutions, and individual practitioners are critical to learn their practical needs from AMR modeling. However, formal reports recording such communications are lacking in scientific journals, which is another factor limiting the generation and use of operational forecasts in real-world settings.

To illustrate the interconnected challenges faced by AMRO forecasting across scales, we use methicillin-resistant *Staphylococcus aureus* (MRSA) as a concrete example (Figure). Several key issues on MRSA forecasting at the facility scale and population scale and across scales are unresolved; one is that the specific data needed for modeling at those different scales are unknown, as is the role of co-selection and competition with methicillin-susceptible *S. aureus* (MSSA) in affecting the dynamics of MRSA. Answering those questions would improve methods to reduce MRSA burden in both community and hospital settings.

**Figure:**
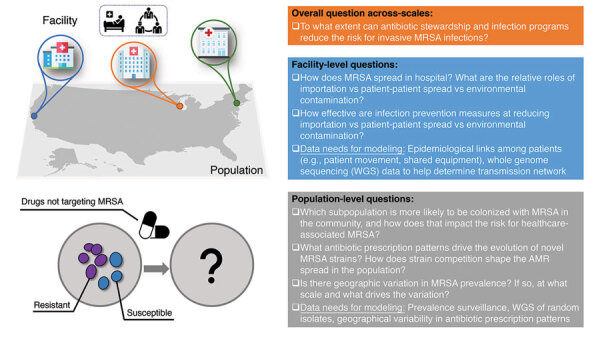
Open questions for predictive modeling of MRSA. Example questions at the facility level, the population level, and across scales are listed. The upper left panel depicts population-level and facility-level MRSA transmission. The lower left panel represents the uncertainty about the roles of co-selection and competition with MSSA in affecting the dynamics of MRSA. MRSA, methicillin-resistant *Staphylococcus aureus*; MSSA, methicillin-susceptible *S. aureus*.

In this perspective, we focus on real-time forecasting of AMROs. A parallel line of research is scenario-based simulations that project AMR infections conditional on postulated changes in prescribed interventions or expected conditions. Previous studies for HIV and tuberculosis control show that such scenario-based projections can substantially affect health policies and save lives (*47*,*48*). For AMR, scenario-based projections should be designed to address practical questions in public health and inform operational policy decision-making in real time, possibly using ensemble approaches that combine multiple models to reflect cross-model variation. Real-time forecasting and scenario-based projections complement each other and should be developed in tandem to control AMR burden and improve human health.

## What Can Be Done Now?

Despite all those challenges, research can still be conducted using currently available data and resources. For instance, the feasibility and utility of real-time forecasting of population-level AMR prevalence could be tested using existing surveillance data. Such an exercise might galvanize the research community to address initial practical questions on forecast design (e.g., What variables should be included? What is an appropriate forecast horizon? How forecast skill be evaluated?).

Increasing the availability of existing data could also accelerate progress. Electronic health records contain a wealth of AMR data, each of which reflects a certain aspect of AMR-related processes. Synthesizing previously siloed datasets into mathematical models can potentially answer scientific questions that are otherwise challenging to address using each dataset separately. Privacy-preserved data sharing across facilities can increase the amount of data for modeling and support the development of generalizable methods. When data sharing is not practical, models and algorithms can be shared, trained, and implemented with defined standards and quality control.

## Future Opportunities

Given existing gaps in forecasting AMR, predictive models are still not mature enough for operational application. To push forward advances in this burgeoning area, several research directions should be prioritized. First, better communication among multiple sectors and stakeholders, including academic researchers, public health agencies, healthcare providers, and the public, will help identify key questions and the needs of end users of predictive models. Developing and applying AMR forecasting will be a collective effort that should address real-world questions in public health and patient care. Second, studies should make better use of existing data and guide the collection of new data that are essential to understand AMR. Investing in consistent surveillance and data collection is of utmost importance for improving understanding of the emergence, spread, and outcomes of AMR. Third, more effective, computationally efficient algorithms are needed to calibrate complex AMR models to multitype and multiscale data. Better interpretability of models can infuse confidence in clinicians when using those tools. Further, research on computational methods that are tailored to AMR prediction could help bridge theoretical models and real-world applications. Fourth, predictive AMR models should be implemented in real-world settings in real time so that operational utility can be assessed by validating real-time operational predictions, as is done for numerical weather predictions. Forecasting skill, including forecast accuracy and uncertainty, should be evaluated to confirm that predictive models can produce useful predictions despite noisy and incomplete data.

In summary, despite lessons learned from recent advances in forecasting for other acute infectious diseases, AMRO prediction has its own set of challenges, including wide and prolonged asymptomatic carriage, longer time scales, continuing evolution due to strain competition and antimicrobial drug use, and poorly observed disease burden. It will be critical to set appropriate expectations for the performance of AMRO predictions and establish sensible criteria for successful forecasting.

## References

[1] World Health Organization. Antimicrobial resistance. 2021 [cited 2022 Jul 18]. https://www.who.int/news-room/fact-sheets/detail/antimicrobial-resistance

[2] Murray CJL, Ikuta KS, Sharara F, Swetschinski L, Robles Aguilar G, Gray A, et al.; Antimicrobial Resistance Collaborators. Global burden of bacterial antimicrobial resistance in 2019: a systematic analysis. Lancet. 2022;399:629–55. 10.1016/S0140-6736(21)02724-0 35065702 PMC8841637

[3] Reich NG, Brooks LC, Fox SJ, Kandula S, McGowan CJ, Moore E, et al. A collaborative multiyear, multimodel assessment of seasonal influenza forecasting in the United States. Proc Natl Acad Sci U S A. 2019;116:3146–54. 10.1073/pnas.1812594116 30647115 PMC6386665

[4] Johansson MA, Apfeldorf KM, Dobson S, Devita J, Buczak AL, Baugher B, et al. An open challenge to advance probabilistic forecasting for dengue epidemics. Proc Natl Acad Sci U S A. 2019;116:24268–74. 10.1073/pnas.1909865116 31712420 PMC6883829

[5] Cramer EY, Ray EL, Lopez VK, Bracher J, Brennen A, Castro Rivadeneira AJ, et al. Evaluation of individual and ensemble probabilistic forecasts of COVID-19 mortality in the United States. Proc Natl Acad Sci U S A. 2022;119:e2113561119. 10.1073/pnas.2113561119 35394862 PMC9169655

[6] Opatowski L, Guillemot D, Boëlle PY, Temime L. Contribution of mathematical modeling to the fight against bacterial antibiotic resistance. Curr Opin Infect Dis. 2011;24:279–87. 10.1097/QCO.0b013e3283462362 21467930

[7] López-Lozano JM, Lawes T, Nebot C, Beyaert A, Bertrand X, Hocquet D, et al.; THRESHOLDS study group. A nonlinear time-series analysis approach to identify thresholds in associations between population antibiotic use and rates of resistance. Nat Microbiol. 2019;4:1160–72. 10.1038/s41564-019-0410-0 30962570

[8] Lawes T, Lopez-Lozano JM, Nebot CA, Macartney G, Subbarao-Sharma R, Dare CR, et al. Effects of national antibiotic stewardship and infection control strategies on hospital-associated and community-associated meticillin-resistant Staphylococcus aureus infections across a region of Scotland: a non-linear time-series study. Lancet Infect Dis. 2015;15:1438–49. 10.1016/S1473-3099(15)00315-1 26411518

[9] Niewiadomska AM, Jayabalasingham B, Seidman JC, Willem L, Grenfell B, Spiro D, et al. Population-level mathematical modeling of antimicrobial resistance: a systematic review. BMC Med. 2019;17:81. 10.1186/s12916-019-1314-9 31014341 PMC6480522

[10] Bonten MJ, Austin DJ, Lipsitch M, Lipsitch M, Lipsitch M. Understanding the spread of antibiotic resistant pathogens in hospitals: mathematical models as tools for control. Clin Infect Dis. 2001;33:1739–46. 10.1086/323761 11595995

[11] Doan TN, Kong DCM, Kirkpatrick CMJ, McBryde ES. Optimizing hospital infection control: the role of mathematical modeling. Infect Control Hosp Epidemiol. 2014;35:1521–30. 10.1086/678596 25419775

[12] Blanquart F. Evolutionary epidemiology models to predict the dynamics of antibiotic resistance. Evol Appl. 2019;12:365–83. 10.1111/eva.12753 30828361 PMC6383707

[13] Davies NG, Flasche S, Jit M, Atkins KE. Within-host dynamics shape antibiotic resistance in commensal bacteria. Nat Ecol Evol. 2019;3:440–9. 10.1038/s41559-018-0786-x 30742105 PMC6420107

[14] Colijn C, Cohen T, Fraser C, Hanage W, Goldstein E, Givon-Lavi N, et al. What is the mechanism for persistent coexistence of drug-susceptible and drug-resistant strains of *Streptococcus pneumoniae?* J R Soc Interface. 2010;7:905–19. 10.1098/rsif.2009.0400 19940002 PMC2871802

[15] Lehtinen S, Blanquart F, Lipsitch M, Fraser C; with the Maela Pneumococcal Collaboration. On the evolutionary ecology of multidrug resistance in bacteria. PLoS Pathog. 2019;15:e1007763. 10.1371/journal.ppat.1007763 31083687 PMC6532944

[16] Blanquart F, Lehtinen S, Lipsitch M, Fraser C. The evolution of antibiotic resistance in a structured host population. J R Soc Interface. 2018;15:20180040. 10.1098/rsif.2018.0040 29925579 PMC6030642

[17] Slayton RB, Toth D, Lee BY, Tanner W, Bartsch SM, Khader K, et al. Vital Signs: estimated effects of a coordinated approach for action to reduce antibiotic-resistant infections in health care facilities—United States. MMWR Morb Mortal Wkly Rep. 2015;64:826–31. 10.15585/mmwr.mm6430a4 26247436 PMC4654955

[18] Smith DL, Levin SA, Laxminarayan R. Strategic interactions in multi-institutional epidemics of antibiotic resistance. Proc Natl Acad Sci U S A. 2005;102:3153–8. 10.1073/pnas.0409523102 15677330 PMC549473

[19] Paul P, Slayton RB, Kallen AJ, Walters MS, Jernigan JA. Modeling regional transmission and containment of a healthcare-associated multidrug-resistant organism. Clin Infect Dis. 2020;70:388–94. 30919885 10.1093/cid/ciz248 PMC6765447

[20] Worby CJ, Jeyaratnam D, Robotham JV, Kypraios T, O’Neill PD, De Angelis D, et al. Estimating the effectiveness of isolation and decolonization measures in reducing transmission of methicillin-resistant *Staphylococcus aureus* in hospital general wards. Am J Epidemiol. 2013;177:1306–13. 10.1093/aje/kws380 23592544 PMC3664336

[21] Cooper BS, Medley GF, Stone SP, Kibbler CC, Cookson BD, Roberts JA, et al. Methicillin-resistant *Staphylococcus aureus* in hospitals and the community: stealth dynamics and control catastrophes. Proc Natl Acad Sci U S A. 2004;101:10223–8. 10.1073/pnas.0401324101 15220470 PMC454191

[22] Bootsma MCJ, Diekmann O, Bonten MJM. Controlling methicillin-resistant *Staphylococcus aureus*: quantifying the effects of interventions and rapid diagnostic testing. Proc Natl Acad Sci U S A. 2006;103:5620–5. 10.1073/pnas.0510077103 16565219 PMC1459403

[23] Pei S, Morone F, Liljeros F, Makse H, Shaman JL. Inference and control of the nosocomial transmission of methicillin-resistant *Staphylococcus aureus*. eLife. 2018;7:e40977. 10.7554/eLife.40977 PMC6298769 30560786

[24] Pei S, Liljeros F, Shaman J. Identifying asymptomatic spreaders of antimicrobial-resistant pathogens in hospital settings. Proc Natl Acad Sci U S A. 2021;118:e2111190118. 10.1073/pnas.2111190118 34493678 PMC8449327

[25] Lee BY, McGlone SM, Wong KF, Yilmaz SL, Avery TR, Song Y, et al. Modeling the spread of methicillin-resistant *Staphylococcus aureus* (MRSA) outbreaks throughout the hospitals in Orange County, California. Infect Control Hosp Epidemiol. 2011;32:562–72. 10.1086/660014 21558768 PMC3388111

[26] Toth DJA, Khader K, Slayton RB, Kallen AJ, Gundlapalli AV, O’Hagan JJ, et al. The potential for interventions in a long-term acute care hospital to reduce transmission of carbapenem-resistant Enterobacteriaceae in affiliated healthcare facilities. Clin Infect Dis. 2017;65:581–7. 10.1093/cid/cix370 28472233 PMC12231108

[27] Knight GM, Davies NG, Colijn C, Coll F, Donker T, Gifford DR, et al. Mathematical modelling for antibiotic resistance control policy: do we know enough? BMC Infect Dis. 2019;19:1011. 10.1186/s12879-019-4630-y 31783803 PMC6884858

[28] Pouwels KB, Muller-Pebody B, Smieszek T, Hopkins S, Robotham JV. Selection and co-selection of antibiotic resistances among *Escherichia coli* by antibiotic use in primary care: An ecological analysis. PLoS One. 2019;14:e0218134. 10.1371/journal.pone.0218134 31181106 PMC6557515

[29] Olesen SW, Barnett ML, MacFadden DR, Brownstein JS, Hernández-Díaz S, Lipsitch M, et al. The distribution of antibiotic use and its association with antibiotic resistance. eLife. 2018;7:e39435. 10.7554/eLife.39435 30560781 PMC6307856

[30] Lehtinen S, Blanquart F, Croucher NJ, Turner P, Lipsitch M, Fraser C. Evolution of antibiotic resistance is linked to any genetic mechanism affecting bacterial duration of carriage. Proc Natl Acad Sci U S A. 2017;114:1075–80. 10.1073/pnas.1617849114 28096340 PMC5293062

[31] Olesen SW, Lipsitch M, Grad YH. The role of “spillover” in antibiotic resistance. Proc Natl Acad Sci U S A. 2020 Nov 2 [Epub ahead of print 10.1073/pnas.2013694117 PMC7682407 33139558

[32] Penders J, Stobberingh EE, Savelkoul PH, Wolffs PF. The human microbiome as a reservoir of antimicrobial resistance. Front Microbiol. 2013;4:87. 10.3389/fmicb.2013.00087 23616784 PMC3627978

[33] Anthony WE, Burnham CD, Dantas G, Kwon JH. The gut microbiome as a reservoir for antimicrobial resistance. J Infect Dis. 2021;223(Suppl 2):S209–13. 10.1093/infdis/jiaa497 33326581 PMC8206794

[34] Relman DA, Lipsitch M. Microbiome as a tool and a target in the effort to address antimicrobial resistance. Proc Natl Acad Sci U S A. 2018;115:12902–10. 10.1073/pnas.1717163115 30559176 PMC6304941

[35] Morley VJ, Woods RJ, Read AF. Bystander selection for antimicrobial resistance: implications for patient health. Trends Microbiol. 2019;27:864–77. 10.1016/j.tim.2019.06.004 31288975 PMC7079199

[36] Tedijanto C, Olesen SW, Grad YH, Lipsitch M. Estimating the proportion of bystander selection for antibiotic resistance among potentially pathogenic bacterial flora. Proc Natl Acad Sci U S A. 2018;115:E11988–95. 10.1073/pnas.1810840115 30559213 PMC6304942

[37] Dahms RA, Johnson EM, Statz CL, Lee JT, Dunn DL, Beilman GJ. Third-generation cephalosporins and vancomycin as risk factors for postoperative vancomycin-resistant enterococcus infection. Arch Surg. 1998;133:1343–6. 10.1001/archsurg.133.12.1343 9865653

[38] National Antimicrobial Resistance Monitoring System for Enteric Bacteria (NARMS). 2020 [cited 2022 Jun 23]. https://www.cdc.gov/narms/index.html

[39] Iskandar K, Molinier L, Hallit S, Sartelli M, Hardcastle TC, Haque M, et al. Surveillance of antimicrobial resistance in low- and middle-income countries: a scattered picture. Antimicrob Resist Infect Control. 2021;10:63. 10.1186/s13756-021-00931-w 33789754 PMC8011122

[40] Cooper BS, Medley GF, Bradley SJ, Scott GM. An augmented data method for the analysis of nosocomial infection data. Am J Epidemiol. 2008;168:548–57. 10.1093/aje/kwn176 18635575 PMC2519111

[41] Thomas A, Redd A, Khader K, Leecaster M, Greene T, Samore M. Efficient parameter estimation for models of healthcare-associated pathogen transmission in discrete and continuous time. Math Med Biol. 2015;32:79–98. 10.1093/imammb/dqt021 24114068

[42] Eyre DW, Laager M, Walker AS, Cooper BS, Wilson DJ; CDC Modeling Infectious Diseases in Healthcare Program (MInD-Healthcare). Probabilistic transmission models incorporating sequencing data for healthcare-associated *Clostridioides difficile* outperform heuristic rules and identify strain-specific differences in transmission. PLOS Comput Biol. 2021;17:e1008417. 10.1371/journal.pcbi.1008417 33444378 PMC7840057

[43] Biggerstaff M, Alper D, Dredze M, Fox S, Fung ICH, Hickmann KS, et al.; Influenza Forecasting Contest Working Group. Results from the centers for disease control and prevention’s predict the 2013-2014 Influenza Season Challenge. BMC Infect Dis. 2016;16:357. 10.1186/s12879-016-1669-x 27449080 PMC4957319

[44] Biggerstaff M, Johansson M, Alper D, Brooks LC, Chakraborty P, Farrow DC, et al. Results from the second year of a collaborative effort to forecast influenza seasons in the United States. Epidemics. 2018;24:26–33. 10.1016/j.epidem.2018.02.003 29506911 PMC6108951

[45] McGowan CJ, Biggerstaff M, Johansson M, Apfeldorf KM, Ben-Nun M, Brooks L, et al.; Influenza Forecasting Working Group. Collaborative efforts to forecast seasonal influenza in the United States, 2015-2016. Sci Rep. 2019;9:683. 10.1038/s41598-018-36361-9 30679458 PMC6346105

[46] Viboud C, Sun K, Gaffey R, Ajelli M, Fumanelli L, Merler S, et al.; RAPIDD Ebola Forecasting Challenge group. The RAPIDD ebola forecasting challenge: Synthesis and lessons learnt. Epidemics. 2018;22:13–21. 10.1016/j.epidem.2017.08.002 28958414 PMC5927600

[47] Blower SM, Gershengorn HB, Grant RM. A tale of two futures: HIV and antiretroviral therapy in San Francisco. Science. 2000;287:650–4. 10.1126/science.287.5453.650 10649998

[48] Blower SM, Small PM, Hopewell PC. Control strategies for tuberculosis epidemics: new models for old problems. Science. 1996;273:497–500. 10.1126/science.273.5274.497 8662538

